# A study of RNA m6A demethylases in oral epithelial dysplasia and oral squamous cell carcinoma

**DOI:** 10.1016/j.jobcr.2022.12.003

**Published:** 2022-12-10

**Authors:** Chatchaphan Udompatanakorn, Pichamon Sriviriyakul, Patrayu Taebunpakul

**Affiliations:** a Department of Oral Surgery and Oral Medicine, Faculty of Dentistry, Srinakharinwirot University, Bangkok, Thailand; b Department of Oral Surgery and Oral Medicine, Faculty of Dentistry, Srinakharinwirot University, Bangkok, Thailand

**Keywords:** RNA demethylase, FTO, ALKBH5, Oral epithelial dysplasia, Oral precancerous lesions, Oral squamous cell carcinoma

## Abstract

**Purpose:**

N6-Methyladenosine (m6A) modification is involved in the tumorigenesis of various cancers. However, the roles of RNA m6A demethylases, fat mass and obesity-associated protein (FTO), and AlkB homolog 5 (ALKBH5) in oral epithelial dysplasia (OED) and oral squamous cell carcinoma (OSCC) remain unclear. This study focuses on FTO and ALKBH5 expression by using immunohistochemistry.

**Material and methods:**

Twenty specimens each of OED, OSCC, and normal oral mucosa (NOM) were included. The expression pattern, the number of positive cells, the cell-staining intensity, and the histochemical scoring (H-score) were examined and analyzed.

**Results:**

In all the OED and OSCC specimens, FTO and ALKBH5 were mainly expressed with moderate to strong staining intensity in the nuclei of the abnormal epithelial cells, respectively. Regarding the NOM, both RNA demethylases showed mild cell staining intensity and was present in 50–60% of the specimens. Interestingly, the percentage of cell positivity, the cell-staining intensity, and the H-score of the FTO and ALKBH5 in NOM, OED, and OSCC were increased, respectively (p < 0.001). There was also a positive correlation between the FTO and ALKBH5 expressions in OSCC (r = 0.62, p = 0.003), but not in the NOM and OED.

**Conclusion:**

These results suggest a possible prognostic role of FTO and ALKBH5 expression in the malignant transformation of OED and tumor progression. Further studies are needed to elucidate the mechanisms underlying the roles of FTO and ALKBH5 in carcinogenesis.

## Graphical abstract

**Figure:**
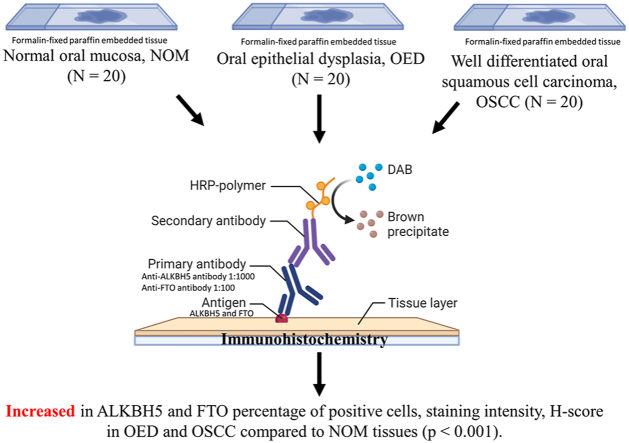

## 1 Introduction

Oral cancer is the sixth most common cancer in the world. Over 90% of oral cancers are oral squamous cell carcinomas (OSCCs).^1^ Most OSCC is preceded by potentially malignant disorders such as oral leukoplakia and oral erythroplakia.^2^ The different degrees of epithelial dysplasia may present in the potentially malignant disorders leading to an increased malignant transformation rate of the lesions.^2^ The gold standard method for OSCC treatment is surgery with or without chemoradiotherapy.^3^ Despite the advances in the detection and treatment of OSCC in the last decade, the five-year survival rates have remained unimproved.^3^ Therefore, finding the novel biomarkers for use as prognostic indicators and targeted therapy for OSCC is essential.^1^^,^^3^

N6-methyladenosine (m6A) modification is the methylation of the nitrogen-6 position of the adenosine bases in the mRNA of eukaryotes and mammalian.^4^ m6A plays important roles in regulating the RNA metabolism, including RNA splicing, stability and translation, embryonic development, and tumorigenesis in various cancers.^4^ m6A modification is a reversible process and its biological functions are controlled by m6A writers or the methyltransferase complex (e.g. methyltransferase-like 3 (METTL3) and METTL14), m6A erasers or demethylases (e.g. fat mass and obesity-associated protein (FTO) and AlkB homolog 5 (ALKBH5)), and m6A readers that recognize m6A methylated transcription (e.g. YT521-B homology (YTH) domain family).^4^^,^^5^

FTO is known to be associated with adipogenesis, obesity, and type 2 diabetes in humans.^4^, ^5^, ^6^ It has been identified as the first RNA demethylase.^4^, ^5^, ^6^ ALKBH5 is physiologically associated with spermatogenesis, ossification, and the innate immunity response in humans.^4^^,^^5^^,^^7^ Recent studies have demonstrated that FTO and ALKBH5 can have an oncogenic or tumor-suppressive role in various cancers.^4^, ^5^, ^6^, ^7^ Additionally, Jin et al. (2022) used The Cancer Genome Atlas (TCGA) database and analyzed the m6A regulatory factors in head and neck squamous cell carcinoma (HNSCC). They reported that half of the expression profile of m6A writers, erasers and readers were dysregulated in HNSCC. Among the 14 m6A regulatory factors, ALKBH5 was significantly elevated.^8^ Another study by Li et al. (2021) has shown that FTO expression was higher in HNSCC than in normal tissues using the data collected from TCGA for analysis.^9^ However, the roles of m6A demethylases in OSCC remain largely unknown.^8^

To our knowledge, few studies have reported about the expression and roles of FTO in OSCC.^9^, ^10^, ^11^ Those studies have shown that higher FTO mRNA and protein expressions were observed in OSCC tissues and cell lines, compared to normal tissues.^9^, ^10^, ^11^ SiRNA knockdown of FTO has been shown to suppress the cellular proliferation, migration, and invasion of OSCC cell lines.^9^, ^10^, ^11^ Regarding ALKBH5 expression, a previous study reported an increase in ALKBH5 in OSCC specimens from chemotherapy non-responders, compared to OSCC specimens from chemotherapy-naive patients.^12^ However, the study did not investigate ALKBH5 expression between OSCC specimens and normal tissues. Moreover, the role of FTO and ALKBH5 in oral precancerous lesions, especially in oral epithelial dysplasia (OED), has never been studied.

Therefore, our study focuses on the expression of FTO and ALKBH5 in normal oral mucosa (NOM), OED (low- and high-grade OED), and well-differentiated OSCC tissues using the immunohistochemical technique. The results of this study may provide a better understanding of the roles of m6A demethylases in OED and OSCC development.

### 1.1 Samples

A retrospective study was conducted between November 2020 and April 2022, using a total of sixty paraffin-embedded tissues, including 20 cases of well-differentiated OSCC, 20 cases of OED (10 cases of high-grade OED and 10 cases of low-grade OED), and 20 cases of NOM (15 cases of healthy gingival tissue and 5 cases of normal epithelium overlying the fibroma of buccal mucosa). The inclusion criteria were paraffin-embedded tissues of OED and OSCC, histologically graded according to the criteria of the World Health Organization^13^; fibroma; and healthy gingival tissues, which were obtained during the surgical treatment of third molars from volunteers who were aged 20 years old or above with signed informed consent. The exclusion criteria were inadequate specimens or specimens without epithelium. An oral pathologist (CU) confirmed the histopathological diagnoses. The clinicopathological data, including gender, age, location, histology (for OED), and size of tumor (for OSCC), were collected from the records. This study was approved by the Ethics Committee for Research on Human Subjects at Srinakharinwirot University, no. SWUEC/X-463/2563 and no. SWUEC/X-031/2565.

### 1.2 Immunohistochemistry

Four-micrometer tissue sections were deparaffinized and rehydrated. The immunohistochemistry was performed using an EnVision kit (Dako Agilent, USA). Antigen retrieval for ALKBH5 and FTO was performed through immersion in a citrate buffer pH 6.0 and Tris-EDTA buffer pH 9.0, respectively, by using a microwave (700 W) for 10 min. The sections were allowed to cool down for 20 min and were then rinsed with tap water for 5 min. Endogenous peroxidase activity was blocked for 30 min. The sections were then washed in a wash buffer. The sections were incubated overnight at 4 °C, with the primary antibodies against ALKBH5 (ab244296, abcam, UK) at a concentration of 1:1000, and against FTO (ab124892, abcam, UK) at a concentration of 1:100. The secondary antibody reagent was then applied to the sections for 30 min at room temperature. The sections were washed with a wash buffer before being incubated with chromogen,3,3I-Diaminobenzidine (DAB) for 5 min. The sections were subsequently rinsed with tap water and counterstained with Mayer's hematoxylin. Finally, the sections were washed, dehydrated, and mounted using the Bio Mount HM mounting medium (Bio-Optica, Italy). Human kidney tissues were taken as a positive control for both antibodies. For negative staining control, the protein block (5% BSA in TBS-T) without primary antibodies was added.

### 1.3 Interpretation of immunostaining

The ALKBH5 and FTO expression were recorded according to the expression patterns, the number of positive cells, the cell-staining intensity, and the histochemical scores (H-score).^14^ The slides were viewed and photographed using a Motic Microscope camera (Motic, China) with a 100x magnification. All the photographs were scored using ImageJ software (NIH, USA). At least 1,000 epithelial cells were counted on each slide. Brown nuclear staining was regarded as positive. The staining intensity was divided into four criteria: 0 = absence of staining; 1 = weak staining; 2 = moderate staining; and 3 = strong staining. The H-score was assessed by multiplying the percentage of cells with staining intensity, which comprised a value from 0 to 300.^14^ The H-score was independently scored by one investigator (PS) and confirmed by the oral pathologist (CU). The inter-investigator calibrations were performed with intraclass correlation coefficient values equal to 0.99. The positive cell-staining of the ALKBH5 and FTO were categorized as high ALKBH5 and FTO expression when the H-score was valued at >150, and low ALKBH5 and FTO expression when the H-score was valued at ≤ 150.

### 1.4 Statistical analysis

GraphPad Prism Version 9 software for Windows (GraphPad Software, USA) was used to perform the statistical analyses. The percentage of positive cells and the H-score among the groups were compared using the Kruskal-Wallis test. The comparisons between the two groups were assessed using Dunn's multiple-comparison test. The scores of the cell-staining intensity among and between the groups was compared using the chi-square test. The differences in the percentage of positive cells and the H-score between low-grade and high-grade OED were calculated using an unpaired *t*-test. The associations between the ALKBH5 and FTO were analyzed by using the correlation coefficient. Additionally, the relationship between the ALKBH5 and FTO expression with the clinicopathological variables were analyzed using Fisher's exact test. The statistical significance was defined as a p value of <0.05.

## 2 Results

### 2.1 Demographic data of the study group

The mean ages of the NOM, OED, and OSCC were 35.1 ± 13.2, 57.7 ± 12.4, and 64.3 ± 14.7 years, respectively. A male predominance was observed in the NOM and OSCC groups, while a female predominance was observed in the OED one. Significant differences involving age and sex distribution among the groups were observed (p < 0.05). In the OED and OSCC groups, the specimens were mostly obtained from the tongue (55%) and the gingiva (50%), respectively. The NOM specimens were mainly taken from the gingiva (75%). In the OSCC group, 55% of cases had a tumor size of 2 cm or less and 45% of the cases had a tumor size of greater than 2 cm, but less than 5 cm. The OED group was histologically graded into 10 cases each of low-grade OED and high-grade OED (Table 1).

**Table 1:** Demographic and other characteristics of the study population.

	NOM No. = 20 (%)	OED No. = 20 (%)	OSCC No. = 20 (%)	p-value
Gender				
Male	12 (60)	8 (40)	16 (80)	0.0357
Female	8 (40)	12 (60)	4 (20)	
Age (years old)				
Mean ± SD	35.1 ± 13.2	57.7 ± 12.4	64.3 ± 14.7	<0.0001
Range	21–64	31–82	41–83	
Site of biopsy				
Gingiva	15 (75)	2 (10)	10 (50)	N/A
Tongue	0 (0)	11 (55)	4 (20)	
Palate	0 (0)	4 (20)	3 (15)	
Buccal mucosa	5 (25)	3 (15)	2 (10)	
Floor of mouth	0 (0)	0 (0)	1 (5)	
OED histopathology				
Mild	–	10 (50)	–	N/A
Moderate to severe	–	10 (50)	–	
OSCC histopathology				
Well-differentiated	–	–	20 (100)	N/A
Moderately to poorly differentiated	–	–	0 (0)	
Size of OSCC				
≤2 cm.	–	–	11 (55)	N/A
>2 - ≤5 cm.	–	–	9 (45)	

SD, standard deviation; N/A, not applicable; NOM, normal oral mucosa; OED, oral epithelial dysplasia; OSCC, oral squamous cell carcinoma. p < 0.05 indicated statistically significant differences.

### 2.2 The expression pattern of ALKBH5 and FTO

**Fig. 1:**
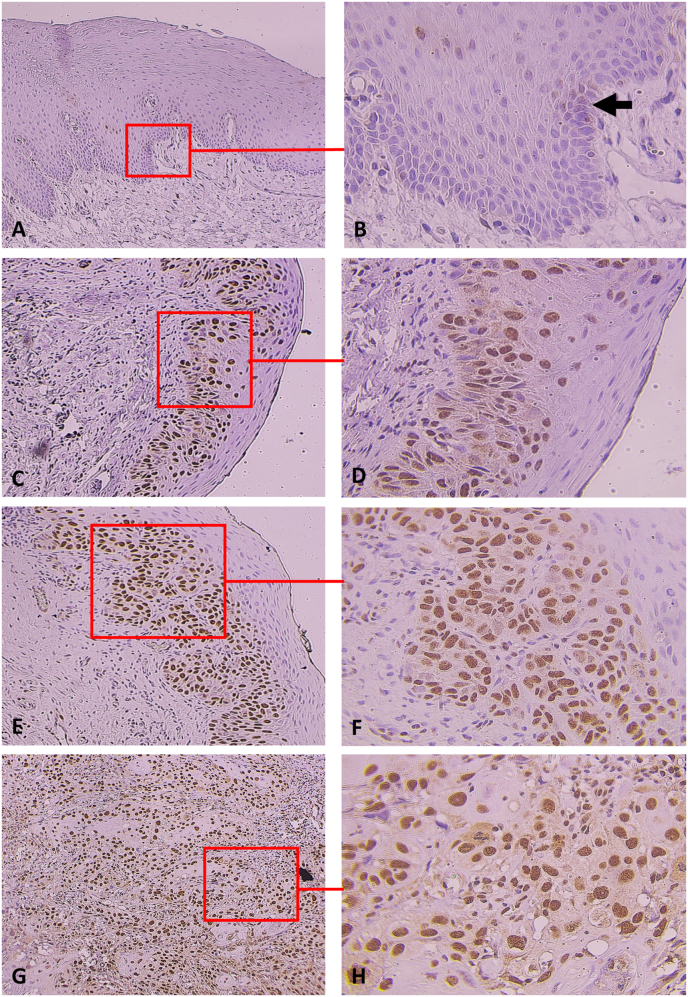
ALKBH5 expression in the representative case of the NOM (A, B), low-grade OED (C, D), high-grade (moderate to severe) OED (E, F), and well-differentiated OSCC (G, H). The expression of ALKBH5 in NOM was found only in the basal and parabasal layers of the squamous epithelium (indicated by an arrow) (A, B). In low-grade OED, ALKBH5 immunostaining was mainly presented in the lower third of the epithelium and partially presented in the spinous layers (C, D). In high-grade OED, ALKBH5 immunostaining was observed in the lower and upper dysplastic epithelium (E, F). In well-differentiated OSCC, ALKBH5 immunostaining was found in the tumor nests (G, H). Original magnification × 100 (A, C, E, G), × 400 (B, D, F, H).

**Fig. 2:**
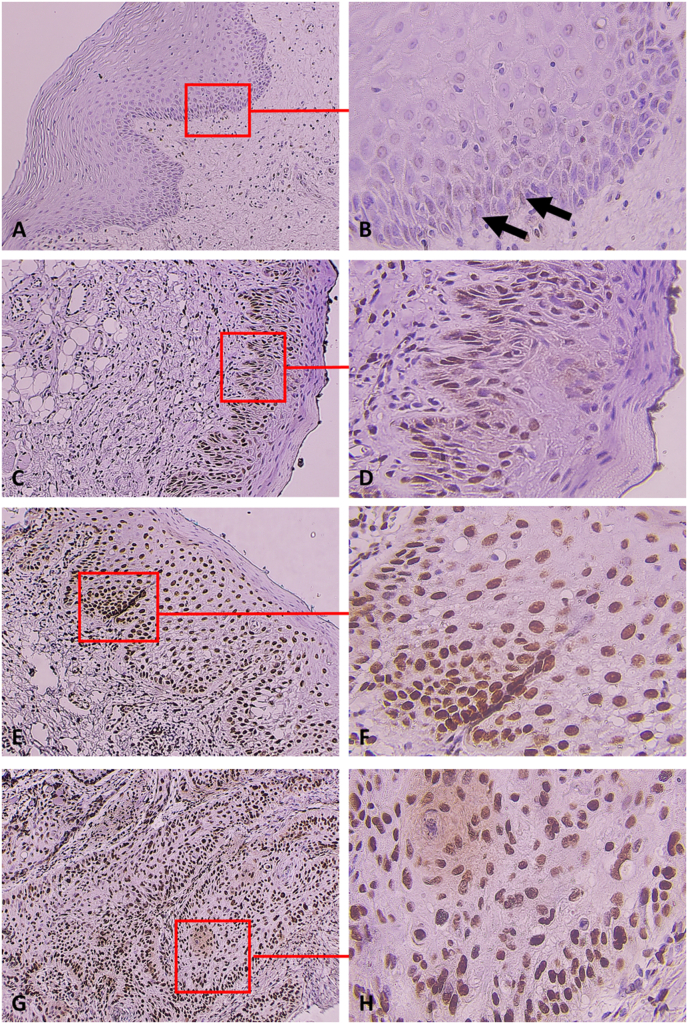
FTO expression in the representative cases of the NOM (A, B), low-grade OED (C, D), high-grade OED (carcinoma in situ) (E, F), and well-differentiated OSCC (G, H). The expression of FTO in the NOM group was limited exclusively to the basal and parabasal epithelial cell layers (indicated by an arrow) (A, B). In low-grade OED, FTO immunostaining was mainly presented in the lower layers of the epithelial cells and partially presented in the spinous layers (C, D). In high-grade OED, FTO immunostaining was observed in the lower and upper dysplastic epithelium (E, F). In well-differentiated OSCC, FTO was observed in the central and peripheral areas of the tumor nests. Original magnification × 100 (A, C, E, G), × 400 (B, D, F, H).

ALKBH5 and FTO immunopositivity were observed only in the nucleus of the samples. For the NOM group, ALKBH5 and FTO expression was detected in 10 (50%) and 12 (60%) cases, respectively, and was restricted to the basal and parabasal layers of the epithelium. For the OED and OSCC groups, all specimens were expressed as ALKBH5 and FTO. The expression of the ALKBH5 and FTO in the OED and OSCC groups were stronger than in the NOM one. In the low-grade OED group, ALKBH5 and FTO expression were detected in the area extending from the lower epithelial compartment to the spinous layer of the epithelium as a focal pattern. In the high-grade OED group, the distributions of ALKBH5 and FTO were widely presented from the lower to the upper layers of the dysplastic epithelium. ALKBH5 and FTO immunostaining in the OSCC group were observed in both the peripheral and central areas of the tumor islands (Fig. 1 and Fig. 2).

### 2.3 The percentage of positive cells, the cell-staining intensity, and the H-scores of the FTO and ALKBH5

The mean percentage of the positive cells of the FTO and ALKBH5 in the NOM, OED, and OSCC groups were significantly increased, respectively (p < 0.001). Regarding the cell-staining intensity, most of the NOM samples (75%) showed a negative to weak FTO and ALKBH5-staining intensity. A moderate to strong FTO and ALKBH5-staining intensity was observed in 85% and 95%, and in 70% and 95%, of the OED and OSCC specimens, respectively. Differences in the FTO and ALKBH5 cell-staining intensity were also observed among the groups (p < 0.001) (Table 2).

**Table 2:** Immunohistochemical findings regarding the FTO and ALKBH5.

	FTO				ALKBH5			
	NOM^a^ No. (%)	OED^b^ No. (%)	OSCC^c^ No. (%)	p-value	NOM^a^ No. (%)	OED^b^ No. (%)	OSCC^c^ No. (%)	p-value
Percentage of positive cells				<0.001^abc^				<0.001^abc^
Mean ± SD				<0.001^ab^				<0.001^ab^
	6.34 ± 9.44	41.19 ± 20.71	82.78 ± 17.92	<0.001^ac^	4.03 ± 5.42	32.99 ± 16.65	72.45 ± 27.27	<0.001^ac^
				0.004^bc^				0.016^bc^
Staining intensity								
Score 0	8 (40)	0 (0)	0 (0)	<0.001^abc^	10 (50)	0 (0)	0 (0)	<0.001^abc^
Score 1	7 (35)	3 (15)	1 (5)		5 (25)	6 (30)	1 (5)	
Score 2	5 (25)	9 (45)	8 (40)		4 (20)	12 (60)	11 (55)	
Score 3	0 (0)	8 (40)	11 (55)		1 (5)	2 (10)	8 (40)	
H-score				<0.001^abc^				<0.001^abc^
Mean ± SD	9.73 ± 14.90	85.35 ± 51.82	188.38 ± 56.71	<0.001^ab^	6.96 ± 10.10	61.04 ± 40.97	162.38 ± 70.75	<0.001^ab^
				<0.001^ac^				<0.001^ac^
				0.008^bc^				0.016^bc^

SD, standard deviation; NOM, normal oral mucosa; OED, oral epithelial dysplasia; OSCC, oral squamous cell carcinoma; H-score, histochemical score. ^a^ = NOM; ^b^ = OED; ^c^ = OSCC; ^a,b,c^ represent comparisons between two or more groups for statistical differences; p < 0.05 indicated statistically significant differences.

Regarding the H-score, FTO expression in the OED (85.35 ± 51.82) and OSCC (188.38 ± 56.71) groups were significantly higher than in the NOM (9.73 ± 14.90) group (p < 0.001) (Table 2). Similarly, the H-scores of the ALKBH5 in the OED (61.04 ± 40.97) and OSCC (162.38 ± 70.75) were significantly greater than in the NOM (6.96 ± 10.10) group (p < 0.001) (Table 2). There was also a statistically significant difference in the H-score of the FTO and ALKBH5 between the OED and OSCC (p = 0.008 and p = 0.016, respectively) (Table 2).

The mean percentage of FTO positive cells in the high-grade OED group (50.04 ± 24.10) was greater than in the low-grade OED (32.33 ± 12.26). However, there was no statistically significant difference between the groups (p = 0.05). Likewise, the mean percentage of positive cells of ALKBH5 in high-grade OED (36.27 ± 18.19) was slightly higher than in the low-grade OED (29.70 ± 15.16), but was not statistically significant. The cell-staining intensity and the H-score of the FTO and ALKBH5 also showed no statistically significant difference between the low-grade and high-grade OED (p = 0.75 and p = 0.53, and p = 0.14 and p = 0.32 respectively; see Supplementary Table 1).

### 2.4 Correlations between the ALKBH5 and FTO expression in NOM, OED, and OSCC groups

There was a moderately positive correlation^15^ between the immunohistochemical expression of the ALKBH5 and FTO in the OSCC samples (r = 0.62, p = 0.003). However, no correlation was observed between the immunohistochemical expression of the ALKBH5 and FTO in the OED and NOM samples (r = 0.05, p = 0.83 for both groups) (Table 3).

**Table 3:** Correlations between the expression of FTO and ALKBH5 in each test group.

	ALKBH5		
FTO	NOM	R	0.05
		p-value	0.83
	OED	R	0.05
		p-value	0.83
	OSCC	R	0.62
		p-value	0.003

NOM, normal oral mucosa; OED, oral epithelial dysplasia; OSCC, oral squamous cell carcinoma. p < 0.05 indicated statistically significant differences.

### 2.5 Relationships between the ALKBH5, FTO, and clinicopathological features in the OED and OSCC groups

There was no correlation between the expression of ALKBH5 and FTO with gender, age, the site of the lesions, the tumor size of the OSCC, or the histology of the OED (low-grade and high-grade OED) (p > 0.05; See Supplementary Tables 2 and 3).

## 3 Discussion

The modification of the m6A plays a crucial role in the RNA metabolism and in regulating various physiological and pathological processes.^4^ Emerging evidence has indicated that m6A demethylases are involved in the tumorigenesis in many types of cancer, including OSCCs.^4^^,^^5^ However, the possible role of m6A demethylases in OED has not previously been investigated. In the current study, we attempted to assess and compare the m6A demethylases’ immunohistochemical expression in the NOM, OED, and OSCC groups. In accord with previous studies on OSCC,^12^ laryngeal and esophageal squamous cell carcinoma,^16^^,^^17^ and gastric adenocarcinoma tissues,^18^ we observed nuclear ALKBH5 localization in the OED and OSCC samples. We also found nuclear FTO expression in the OED and OSCC, which corresponds with the previous studies on OSCC,^9^^,^^10^ hypopharyngeal,^19^ and cervical squamous cell carcinoma.^20^ In addition, we found that the percentage of positive cells, the cell-staining intensity, and the H-scores of the ALKBH5 and FTO were significantly increased in the OSCC, compared to the NOM samples. In agreement with our results, Li et al. (2022),^16^ and Jin et al. (2022)^8^ showed that ALKBH5 immunohistochemical expression was increased in the laryngeal and head and neck squamous cell carcinoma tissues, compared to the normal tissues. Wang et al. (2021) also reported that the ALKBH5 and FTO mRNA levels were increased in the OSCC tissues, compared to the normal tissues.^10^ These results suggest the roles of FTO and ALKBH5 in OSCC.

A previous study reported that a knockdown of ALKBH5 significantly increased the percentage of the tumor cells in the G0/G1 phase, along with the upregulated p21 protein expression, but suppressed cell proliferation and migration in the esophageal squamous cell carcinoma cell line.^17^ Zhou et al. (2018) and Zhang et al. (2021) reported that FTO was highly expressed and co-localized with the proliferation marker ß-catenin in the cervical and hypopharyngeal squamous cell carcinoma, respectively.^19^^,^^20^ A knockdown of FTO decreased the proliferation, migration, and expression of epithelial-mesenchymal transition (EMT) protein (Sox2, Slug, and ZEB1) of the OSCC and hypopharyngeal squamous cell carcinoma cell lines.^9^, ^10^, ^11^^,^^19^ The results suggest a role of the ALKBH5 in tumor-cells proliferation and the involvement of FTO in the proliferation and EMT process of the dysplastic cells in the OSCC, esophageal, and hypopharyngeal squamous cell carcinoma. These data further suggest that alteration of the m6A demethylases, FTO, and ALKBH5 are involved in oral epithelium malignant transformation of OSCC.

Jing et al. (2019) reported that a marker of proliferation, Ki-67 expression was increased with the progression of dysplasia in oral mucosal tissues. The expression of Ki-67 was shown to be the highest in OSCC.^21^ Knockdown of ALKBH5 in Cal27 OSCC cell line led to inhibition of tumor growth and decreased expression of Ki-67.^8^ These results suggest that ALKBH5 may have an influence on Ki-67 expression and cell proliferation. Additionally, increased Yes1-associated transcription regulator (YAP1) expression was reported in OED and OSCC when compared to normal tissues.^22^ The expression of YAP1 was shown to be associated with the disease severity and may be a strong driver of OSCC progression.^22^ Previous research has shown that FTO can affect the stability of YAP1.^11^ Thus, these data support the roles of ALKBH5 and FTO in the regulation of oral cancer progression.

Recent studies have described the molecular mechanism of the role of m6A demethylases in the OSCC. FTO promoted OSCC tumorigenesis by enhancing the eIF4G1 stability, thereby decreasing the autophagic process and inducing tumor occurrence.^10^ Li et al. (2022) reported that reduction of FTO increased YAP1 m6A modification, resulting in the degradation of YAP1 mRNA, which is known to promote the development and progression of OSCC.^11^ Shriwas et al. (2020) reported that the ALKBH5 demethylates FOXM1 and NANOG mRNA results in enhanced FOX1 and NANOG expression and contributes to chemoresistance in the OSCC cells.^12^

Regarding OED, our study demonstrates that FTO and ALKBH5 were expressed in all OED samples, especially in abnormal epithelial cells. There was no report on the expression of FTO and ALKBH5 in OED before ours. However, one study reported a greater expression of mRNA expression of FTO in high-grade cervical squamous intraepithelial lesions than in normal tissues.^20^ Those results are similar to ours involving a higher expression of m6A demethylases, FTO, and ALKBH5 in OED, which has the potential to develop into oral cancer. Nevertheless, the molecular mechanism of the role of m6A demethylases in OED remains unknown. Further studies are needed to investigate the molecular mechanism involved in the role of m6A demethylases in OED pathogenesis.

Two studies investigated the co-expression of ALKBH5 and FTO in cancers. Wang et al. (2021) reported that the mRNA level of ALKBH5 and FTO were increased in the OSCC tissues, in contrast to normal tissue. However, the correlations between the two genes were not investigated.^10^ Strick et al. (2021) reported that mRNA expression of ALKBH5 and FTO were significantly downregulated in clear renal cell carcinoma, compared to normal renal tissue. The Spearman's rank correlation test showed a significant correlation between the two genes (p < 0.001).^23^ In our study, we observed a positive correlation between ALKBH5 and FTO protein expression in the OSCC (r = 0.62, p = 0.003), but not in the OED and NOM samples, which suggests that the dysregulation of both m6A demethylases may be involved in the pathogenesis of OSCC.

Increased FTO expression has been associated with a larger tumor size, a higher TNM stage and grade, and a shorter survival time in OSCC patients.^11^ To our knowledge, no previous study has investigated the relationship between ALKBH5 expression and the clinicopathological features in OSCC. We investigated the associations between ALKBH5 and FTO expression with the clinicopathological characteristics. No correlations were observed. This may be attributable to the limited sample size of our study. So, additional studies with larger samples are needed to confirm our preliminary results. There are also other limitations in this study. One of them is the wide differences in mean age and the site of biopsy among groups. Due to ethical considerations, most of the NOM specimens were normal tissues from the third molar impaction site of young patients. Another limitation found is the absence of some clinical parameters such as disease duration and risk factors that are based on chart reviews and cannot be analyzed.

Finally, our previous studies demonstrated that the nuclear expression of methyltransferase-like 3 (METTL3), which is one of the m6A writer, was increased from the NOM to the low-grade and high-grade OED and OSCC samples.^24^ It is possible that the increased expression of m6A demethylases in OED and OSCC observed in our study may have resulted from true dysregulation of the m6A demethylases or compensation for the high expression of METTL3 in the OED and OSCC. These results suggest the important roles of m6A modification in OSCC.

## 4 Conclusion

The expression of ALKBH5 and FTO was gradually increased from the NOM to the OED to the well-differentiated OSCC. The expression of ALKBH5 was positively correlated with FTO expression in OSCC, but not in the NOM and OED. These results suggest that m6A demethylases might be involved in the OED and OSCC pathogenesis. The data from this study may be utilized as an initial step to establish a biomarker for detecting the malignant transformation of OED. Further studies are required to investigate the molecular mechanism of the role of m6A demethylases in oral tumorigenesis.

## Funding

This work was supported by The Dental Hospital, Faculty of Dentistry, Srinakharinwirot University, Bangkok, Thailand [grant number 294/2564].

## Declaration of competing interest

The authors declare that they have no conflicts of interest.
