# Supplementary material for: A study of RNA m6A demethylases in oral epithelial dysplasia and oral squamous cell carcinoma
Source: J Oral Biol Craniofac Res. 2022 Dec 10;13(2):111–6. doi: 10.1016/j.jobcr.2022.12.003 (PMC9792536; doi:10.1016/j.jobcr.2022.12.003)
Supplement: Multimedia component 1 [file mmc1.docx]

**Supplementary Table 1:** The immunohistochemical expression of FTO and ALKBH5 in low-grade OED and high-grade OED

| **Antibody** | **Evaluation** | **OED** | | **p-value** |
| --- | --- | --- | --- | --- |
|  |  | **Low-grade**  **N (%)** | **High-grade**  **N (%)** |  |
| **FTO** | No. of positive cases | 20 (100) | 20 (100) | N/A |
|  | Percentage of positive cells (Mean$\pm$SD) | 32.33$\pm$12.26 | 50.04$\pm$24.10 | 0.05 |
|  | Intensity  score 0  score 1  score 2  score 3 | 0 (0)  1 (10)  5 (50)  4 (40) | 0 (0)  2 (20)  4 (40)  4 (40) | 0.75 |
|  | H-score (Mean$\pm$SD) | 70.20$\pm$31.08 | 100.50$\pm$64.74 | 0.53 |
| **ALKBH5** | No. of positive cases | 20 (100) | 20 (100) | N/A |
|  | Percentage of positive cells (Mean$\pm$SD) | 29.70$\pm$15.16 | 36.27$\pm$18.19 | 0.39 |
|  | Intensity  score 0  score 1  score 2  score 3 | 0 (0)  4 (40)  6 (60)  0 (0) | 0 (0)  2 (20)  6 (60)  2 (20) | 0.14 |
|  | H-score (Mean$\pm$SD) | 51.64$\pm$29.75 | 70.44$\pm$49.61 | 0.32 |

N/A = not available; OED = oral epithelial dysplasia. p < 0.05 indicated statistically significant differences.

**Supplementary Table 2:** Correlations of the FTO expression and clinicopathologic data in the OED and OSCC

| **Clinicopathological features** | **OED** | | **p-value** | **OSCC** | | **p-value** |
| --- | --- | --- | --- | --- | --- | --- |
|  | **Low FTO expression**  **N (%)** | **High FTO expression**  **N (%)** |  | **Low FTO expression**  **N (%)** | **High FTO expression**  **N (%)** |  |
| **Age (years old)**  $\leq$ 60  > 60 | 14 (70)  4 (20) | 1 (5)  1 (5) | 0.44 | 3 (15)  2 (10) | 6 (30)  9 (45) | 0.61 |
| **Gender**  Male  Female | 7 (35)  11 (55) | 1 (5)  1 (5) | 1.0 | 5 (25)  0 (0) | 11 (55)  4 (20) | 0.53 |
| **Site of lesions**  Gingiva  Buccal mucosa  Tongue  Hard palate  Floor of mouth | 1 (5)  3 (15)  11 (55)  3 (15)  0 (0) | 1 (5)  0 (0)  0 (0)  1 (5)  0 (0) | 0.54 | 3 (15)  0 (0)  2 (10)  0 (0)  0 (0) | 7 (35)  2 (10)  2 (10)  3 (15)  1 (5) | 0.49 |
| **Size of OSCC**  <2 cm.  >2 - <5 cm. | N/A  N/A | N/A  N/A | N/A | 4 (20)  1 (5) | 7 (35)  8 (40) | 0.31 |
| **Histology of OED**  Low-grade OED  High-grade OED | 10 (50)  8 (40) | 0 (0)  2 (10) | 0.47 | N/A  N/A | N/A  N/A | N/A |

N/A = not available. p< 0.05 indicated statistically significant differences.

**Supplementary Table 3:** Correlations of the ALKBH5 expression and clinicopathologic data in the OED and OSCC

| **Clinicopathological features** | **OED** | | **p-value** | **OSCC** | | **p-value** |
| --- | --- | --- | --- | --- | --- | --- |
|  | **Low ALKBH5 expression**  **N (%)** | **High ALKBH5 expression**  **N (%)** |  | **Low ALKBH5 expression**  **N (%)** | **High ALKBH5 expression**  **N (%)** |  |
| **Age (years old)**  $\leq$ 60  > 60 | 15 (75)  4 (20) | 0 (0)  1 (5) | 0.25 | 4 (20)  2 (10) | 5 (25)  9 (45) | 0.33 |
| **Gender**  Male  Female | 8 (40)  11 (55) | 0 (0)  1 (5) | 1.0 | 6 (30)  0 (0) | 10 (50)  4 (20) | 0.26 |
| **Site of lesions**  Gingiva  Buccal mucosa  Tongue  Hard palate  Floor of mouth | 2 (10)  3 (15)  11 (55)  3 (15)  0 (0) | 0 (0)  0 (0)  0 (0)  1 (5)  0 (0) | 0.17 | 2 (10)  0 (0)  4 (20)  0 (0)  0 (0) | 8 (40)  2 (10)  0 (0)  3 (15)  1 (5) | 0.68 |
| **Size of OSCC**  <2 cm.  >2 - <5 cm. | N/A  N/A | N/A  N/A | N/A | 5 (25)  1 (5) | 6 (30)  8 (40) | 0.15 |
| **Histology of OED**  Low-grade OED  High-grade OED | 10 (50)  9 (45) | 0 (0)  1 (5) | 1.0 | N/A  N/A | N/A  N/A | N/A |

N/A = not available. p< 0.05 indicated statistically significant differences.
